# Clinical trial for evaluation of *Ricinus communis* and sodium hypochlorite as denture cleanser

**DOI:** 10.1590/1678-7757-2016-0222

**Published:** 2017

**Authors:** Maurício Malheiros BADARÓ, Marcela Moreira SALLES, Vanessa Maria Fagundes LEITE, Carolina Noronha Ferraz de ARRUDA, Viviane de Cássia OLIVEIRA, Cássio do NASCIMENTO, Raphael Freitas de SOUZA, Helena de Freitas de Oliveira PARANHOS, Cláudia Helena SILVA-LOVATO

**Affiliations:** 1 Universidade de São Paulo, Faculdade de Odontologia de Ribeirão Preto, Departamento de Materiais Dentários e Prótese, Ribeirão Preto, SP, Brazil

**Keywords:** Denture, Biofilms, Candidiasis, Disinfection

## Abstract

The development of opportunistic infections due to poor denture hygiene conditions justified the search for effective hygiene protocols for controlling denture biofilm.

**Objective:**

This study evaluated *Ricinus communis* and sodium hypochlorite solutions in terms of biofilm removal ability, remission of candidiasis, antimicrobial activity, and participant satisfaction.

**Material and Methods:**

It was conducted a controlled clinical trial, randomized, double-blind, and crossover. Sixty-four denture wearers with (n=24) and without candidiasis (n=40) were instructed to brush (3 times/day) and immerse their dentures (20 min/day) in different storage solutions (S1 / S2: 0.25% / 0.5% sodium hypochlorite; S3: 10% *R. communis*; S4: Saline).The trial period for each solution was seven days and a washout period of seven days was used before starting the use of another solution. The variables were analyzed at baseline and after each trial period. The biofilm of inner surfaces of maxillary dentures was disclosed, photographed, and total and dyed areas were measured (Image Tool software). The percentage of biofilm was calculated. Remission of candidiasis was assessed by visual scale and score were attributed. Antimicrobial activity was assessed by the DNA-Checkerboard hybridization method. Patient satisfaction was measured using a questionnaire.

**Results:**

S1 (4.41±7.98%) and S2 (2.93±5.23%) were more effective then S3 (6.95±10.93%) in biofilm remotion(P<0.0001). All solutions were different from the control (11.07±11.99%). S3 was the most effective solution in remission of candidiasis (50%), followed by S1 (46%). Concerning antimicrobial action, S1/S2 were similar and resulted in the lowest microorganism mean count (P=0.04), followed by S3. No significant differences were found with patient’s satisfaction.

**Conclusions:**

10% *R. communis* and 0.25% sodium hypochlorite were effective in biofilm removal, causing remission of candidiasis and reducing the formation of microbial colonies in denture surfaces. All solutions were approved by patients.

## Introduction

Complete denture is a potential microbial reservoir, which can lead to infections and inflammatory diseases. Several studies have examined the development of microbial colonies in dentures and in the supporting soft and hard oral tissues^22^ ^,^ ^24^ ^,^ ^25^ ^,^ ^28^. Species commonly found in the oral microbiota of healthy individuals can cause chronic atrophic candidiasis and systemic diseases such as bacterial endocarditis, intestinal infection, chronic obstructive pulmonary disease and aspiration pneumonia^2^ ^,^ ^21^.

Correct denture hygiene is essential to reduce or eliminate pathogens^15^ ^,^ ^22^ ^,^ ^23^ and establish an environment suitable for the growth of beneficial oral microbiota. Studies have shown that a combination of mechanical and chemical cleaning is efficient in the maintenance of denture hygiene^7^ ^,^ ^22^.

Among chemical solutions, sodium hypochlorite (1% and 0.5% NaClO) is the most commonly used and shows good bactericidal and fungicidal properties^15^ ^,^ ^22^ ^,^ ^23^. However, these solutions may adversely affect physical and mechanical properties of the denture^4^ ^,^ ^17^. In addition, the unpleasant taste and odor of NaClO may cause some discomfort for patients, although there aren’t studies that have evaluated the extent of this problem. These aspects may influence the acceptance of antiseptic solutions by denture wearers and, therefore, their usage on a regular basis could be lower than shown in short-term trials^29^. Therefore, studies using lower concentrations are needed.

The method chosen for home prosthetic care should be effective in removing organic and inorganic debris, exhibit fungicidal and bactericidal properties, be compatible with the structural material of the prosthesis, be non-toxic to users, have low cost, and be easy to handle. Since most of the current methods used for denture hygiene do not present all these characteristics, numerous studies have been conducted in an attempt to find an optimal protocol^2^ ^,^ ^4^ ^,^ ^7^ ^,^ ^10^ ^,^ ^16^ ^,^ ^19^ ^,^ ^20^ ^,^ ^22^ ^-^ ^24^ ^,^ ^26^ ^,^ ^27^ ^,^ ^29^.

The *R. communis* solution has been studied as a potential denture cleaner, since it acts as a detergent and has antimicrobial properties. Moreover, it does not have toxic effects on oral tissues^2^ ^,^ ^9^ ^,^ ^18^ ^-^ ^20^. *R. communis* derives from the castor plant (*Ricinus communis*; division *Magnoliophyta*, class *Magnoliopside*, sub-class *Rosidae*, order *Euforbiales*, family *Euforbiaceae*), which is a vegetable native to the Middle East and the northeastern Africa, but is commonly found in tropical climate areas such as Brazil^11^ ^,^ ^20^. The presence of a hydroxyl group, a single point of unsaturation and a carboxyl group – three highly reactive functional groups in the ricinoleic acid present in the castor oil composition – give *R. communis* important oil-chemical potential. It may be subjected to various chemical processes to obtain by-products used in the pharmaceutical and cosmetic industry, in the production of lubricants, polymers, biodiesel^11^ ^,^ ^12^, and more specifically in medical and dental products. Although a few studies have focused on the use of *R. communis* in complete dentures, the available results are promising^2^ ^,^ ^4^ ^,^ ^19^ ^,^ ^20^ ^,^ ^22^ ^,^ ^23^, particularly at a concentration of 10%^10^ ^,^ ^22^ ^,^ ^23^. However, although controlled clinical trials assess this oil’s ability to remove biofilm, its antimicrobial properties and patient acceptability are inconclusive, and call for further investigation.

Thus, the aim of this clinical study was to evaluate the effectiveness of 10% *Ricinus communis* and 0.25% NaClO solution as denture cleaning agents. The properties assessed include the ability to remove biofilm, to reduce candidiasis, as well as antimicrobial properties and patient satisfaction. Results were compared with 0.5% sodium hypochlorite and saline. The first null hypothesis was that 10% *R. communis*, 0.25% sodium hypochlorite and 0.5% sodium hypochlorite denture cleansers would have the same ability to remove biofilm, to cause remission of candidiasis, as well as the same antimicrobial action. The second null hypothesis was that immersion in 10% *R. communis* would have the same acceptance as saline by the patients.

## Methodology

This protocol was approved by the institutional Ethics Committee (CAAE-0013.0.138.000-07) and registered at ClinicalTrials.gov (NC T02407834; U.S. National Institutes of Health). Regular patients from Ribeirão Preto Dental School were invited to participate. Inclusion criteria were: having good general health and motor coordination; wearing conventional maxillary dentures fabricated with heat-activated acrylic resin and in use for 5 to 10 years; and presenting biofilm in the inner surface of dentures (Additive index^1^). Exclusion criteria were: systemic diseases known to foster the growth of *Candida* (e.g., uncontrolled diabetes; immunosuppressive disorders; anemia; xerostomia); use of antibiotics, antifungal agents or corticosteroids; having received chemotherapy or radiotherapy in the last four weeks prior to enrollment in the study. Evidences for denture adaptation problems, the need for reline, repair, or a fractured denture also led to the exclusion of the participant.

Variables of quantitative response were effectiveness of biofilm removal, remission of candidiasis and antimicrobial action. As a qualitative variable, the acceptance of the solutions by the participants was analyzed. Participants were instructed to brush their dentures three times a day (after breakfast, lunch, and dinner) with a specific brush (Bitufo^®^ , Itupeva, SP, Brazil) and neutral liquid soap (Pleasant, Perol Commercial and Industrial Ltda., Ribeirão Preto, SP, Brazil), and to soak the dentures for 20 min, once a day, in 200 mL of the following solutions: S1: 0.25% sodium hypochlorite (Inject Center, Ribeirão Preto, SP, Brazil); S2: 0.5% sodium hypochlorite (Inject Center); S3: 10% *R. communis* oil solution (Institute of Chemistry, University of São Paulo, São Carlos, SP, Brazil); and S4: 0.85% saline solution (control; sodium chloride P.A.; Labsynth Laboratory Products Ltda., Diadema, SP, Brazil). All participants used each solution for seven days in a random sequence (cross-over). Following each period of use, there was a 1-week washout period during which the patients used the specific brush and neutral liquid soap to clean their dentures, in order to eliminate the residual effect of previous treatment (carry over effect)^22^. Participants were instructed to rinse dentures before insertion into the oral cavity and keeping the dentures immersed in water overnight.

For the blinding of involved parts, the products were distributed in unidentified vials (solutions) and delivered without identification to participants, as follows: Researcher P1 obtained a list of random numbers (Excel 2013, Microsoft Brazil, Sao Paulo, SP, Brazil), corresponding to the possible sequences of treatments. All possible sequences had the same probability of being assigned. Researcher P2 received the random numbers and distributed the products to the participants according to the codes. Researcher P3 provided the hygiene instructions and applied the questionnaire. Researchers P4 and P5 were responsible for the take of the dentures, biofilm staining, and subsequent total biofilm elimination. Researchers P6 and P7 obtained the photographs of the dentures, collected the biofilm, and processed it by DNA-Checkerboard method. Researcher P8 conducted biofilm quantification, tabulated the variables, and forwarded the data to researcher P9, who performed the statistical analysis.

### Biofilm quantification

Baseline conditions were recorded for all participants. The intaglio surfaces of the upper dentures were dyed (1% neutral red) and photographed (Canon EOS Digital Rebel EF-S 18-55; Canon MR-14 EX flash, Canon Inc., Tokyo, Japan) with the camera fixed on a stand (CS-4 Copy Stand, Testrite Inst. Co., Inc., Newark, NJ, USA), maintaining a standard film-object distance and controlling exposure time. Images were transferred to a computer, and total surface and stained areas were measured (Software ImageTool 3.0, UTHSC, San Antonio, USA). Biofilm percentage was calculated by the biofilm/total surface area ratio of the denture multiplied by 100^16^ ^,^ ^26^. Thereafter, the biofilm on the denture surface was removed by a researcher (P4 and P5) using a brush with neutral liquid soap. All participants received cleaned dentures at the start of the experimental period. After each experimental period, the intaglio surfaces of the dentures were dyed, and the disclosed biofilm was photographed and analyzed, as previously described.

### Candidiasis assessment

The palatal mucosa of the participants with candidiasis was photographed with the camera focused on the mid-palatal raphe region, with adequate visualization of the entire region, which includes the incisive papilla until the right and left tuberosity. Images were obtained at baseline after seven days of each intervention and after washout periods. Images were transferred to a computer and the Prosthodontic Tissue Index^5^ was applied following scores: “0”(excellent): normal tissue, pink surface, with normal vascularization and appearance; “1” (satisfactory): reddish inflamed mucosa with areas of focal hyperemia, but generally normal appearance; “2” (poor): reddish mucosa with multiple hyperemic areas and widespread shiny surface; “3” (unsatisfactory): markedly red mucosa with or without focal hyperemia, shiny surface and granular inflammation.

### Participant satisfaction

Participants’ satisfaction was measured by the following questions: Q1) Does the product used this week cleaned your prosthesis?; Q2) What is your perception about the smell of the product?; Q3) Did the product leave any taste on your denture?; Q4) Was the product easy to use?; Q5) Would you use the product daily?; Q6) Would you recommend this product to a friend? The questions were answered on a 0–10 scale, in which “0” was the worst possible (most negative) answer and “10” the best possible (most positive) answer.

### Antimicrobial action

DNA-Checkerboard hybridization method was used to assess antimicrobial effect of the solutions^13^. Biofilm was collected from the inner surface of the dentures (incisive papilla, left and right tuberosity regions, regions with the highest propensity to biofilm accumulation) with a sterile microbrush at baseline and after seven days of each treatment. The active tips of the microbrushes were individually inserted into microtubes containing 150 µL of buffer TE (10 mM Tris-HCl, 1 mM EDTA, pH 7.6), followed by addition of 150 µL of 0.5 M NaOH to cause cell lysis.

**Figure 1:**
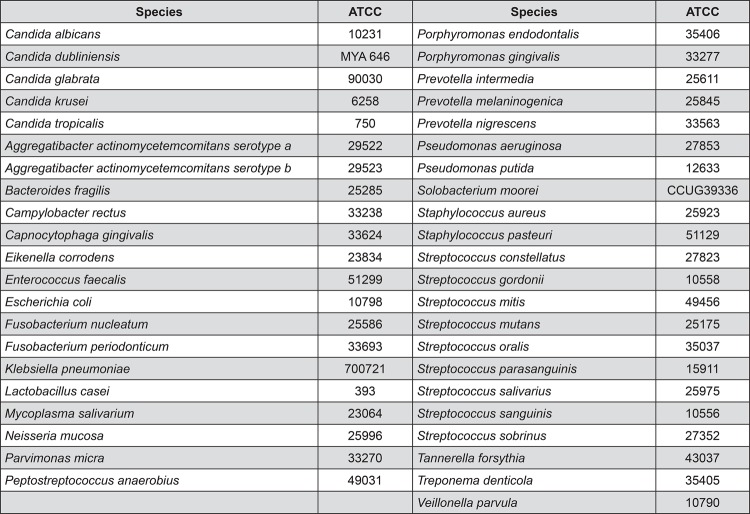
Investigated microorganisms

In short, DNA clinical samples were collected, denatured, precipitated, applied in individual lanes, and fixed onto nylon membranes. For standard samples, mixtures of genomic DNA comprising 10^5^ or 10^6^ microbial cells of each analyzed species were assembled, denatured, precipitated and applied into two control slots. Membranes were pre-hybridized (60°C, 2 h) in a hybridization buffer consisting of NaCl at 0.5 M and blocking reagent at 0.4% (w/vol). Thereafter, membranes received specific aliquots of labeled, whole genomic probes of the proposed target species and hybridized overnight at 60°C under gentle agitation. On the following day, membranes were washed twice in primary wash buffer (65°C, 30 min) and twice in a secondary wash buffer (at room temperature for 15 min). After washing, hybrids were directly detected by chemiluminescence using the Gene Images CDP-Star Reagent (GE Healthcare, UK). Exposure of the membrane to ECL Hyperfilm-MP (GE Healthcare, UK) for 30 min enabled the detection of hybridization. Images on the hyperfilm were digitized and analyzed with the use of TotalLab Quant analysis software (TotalLab Life Science Analysis Essentials; Newcastle upon Tyne). This software translates pixel intensity into amount of microbial cells by comparing samples with standard reference lanes on the membrane. Forty three target species were analyzed, including pathogens associated with denture stomatitis and periodontal disease (Figure 1).

### Sample size and statistical analysis

Sample size was defined according to a previous cross-over trial^16^. That trial used similar outcome assessment methods and found differences in a sample of 36 participants. Therefore, this study enrolled 76 participants, which would allow for possible withdrawals and losses.

The efficacy of the denture cleaning solutions in removing biofilm was analysed using a two-way ANOVA and Tukey’s test (p<0.05). For remission of candidiasis, data were analyzed using multinomial logistic regression. The candidiasis scores from baseline and washout periods were considered as co-variables and candidiasis after treatment was treated as a 4-points ordinal scale. The participants’ satisfaction questionnaire was adjusted by logistic regressions. The correlation structure adopted for this analysis had composite symmetry. Antimicrobial effect was analyzed as presence or absence of inflammation for each solution. First, total microbial count after each treatment was calculated and significant differences between groups were compared using generalized linear models (GLM). In a second analysis, Friedman Test followed by Dunn’s multiple comparisons post-test were used to compare the effect of each solution on individual target species. Differences were considered significant when p<0.05. All tests were performed by the SPSS 21.0 software (SPSS Inc., Chicago, IL, USA).

## Results

**Figure 2:**
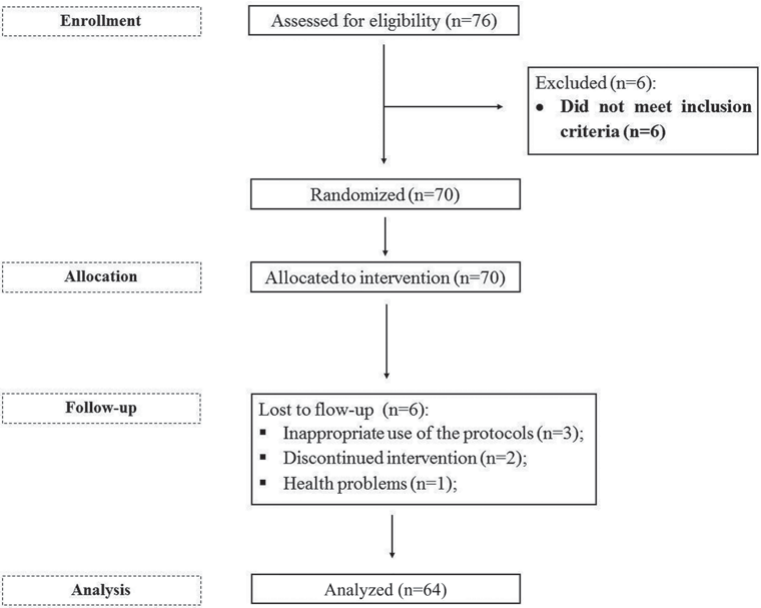
Flowchart of the participants of the study period

The final sample included 24 individuals with oral candidiasis (four men, 20 women; mean age of 69 years) and 40 without oral candidiasis (14 men, 26 women; mean age of 67 years). A flowchart of the participants of the study period is shown in Figure 2. The study was submitted to the Ethics Committee in May 2012 and was carried out from July 2012 to December 2013, being uneventfully completed. The selection of participants took place between July and August 2012.

**Figure 3:**
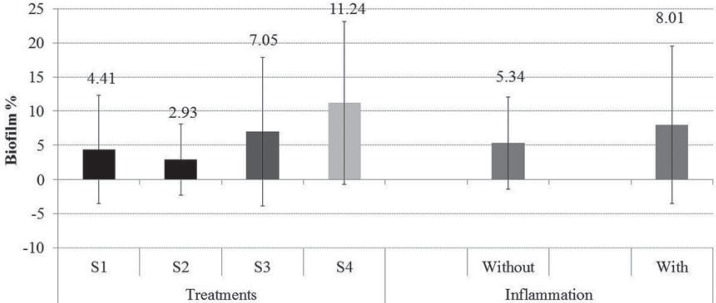
Mean biofilm percentage (±SD) according to the treatments (p=0.0001) and the inflammation (p=0.19). Different colors indicate significant difference

No significant differences were observed in the ability of solutions to remove biofilm between participants with and without inflammation (p=0.19) or interaction between inflammation and solution (p=0.85). The remotion of biofilm was significantly influenced by the solutions (p=0.0001). S1 and S2 solutions yield the lowest percentage of biofilm, followed by S3. S4 had the highest values (Figure 3).

Table 1 shows the frequency (%) of inflammation scores at baseline, washout, and after treatments. A change from score “1” (satisfactory) to “0” (absence) and from score “3” (unsatisfactory) to “2” (regular) was found. Table 2 shows the score movement for each solution. S3 and S1 had the highest percentages of the “improved” and “cured”, being equal to 50% and 46%, respectively.

**Table 1:** Frequency of inflammation score (F) and percentage (%) at baseline/washout and after treatment

Solution	Baseline and Washout						After treatment				
		0	1	2	3	Total	0	1	2	3	Total
S1	F	1	7	12	4	24	6	9	9	0	24
	%	4.2	29.2	50.0	16.7	100	25.0	37.5	37.5	0.0	100
S2	F	3	6	11	4	24	6	7	8	3	24
	%	12.5	25.0	45.8	16.7	100	25.0	29.2	33.3	12.5	100
S3	F	3	8	8	5	24	6	10	7	1	24
	%	12.5	33.3	33.3	20.8	100	25.0	41.7	29.2	4.2	100
S4	F	1	9	11	3	24	0	9	11	4	24
	%	4.2	37.5	45.8	12.5	100	0.0	37.5	45.8	16.7	100
Total	F	8	30	42	16	96	18	35	35	8	96
	%	8.3	31.3	43.8	16.7	100.0	18.8	36.5	36.5	8.3	100.0

**Table 2:** Inflammation rates after treatments

	Worse	Maintained	Improved	Cured	Total
S1	0	13	6	5	24
%	0.0%	54.2%	25.0%	20.8%	100%
S2	2	15	3	4	24
%	8.3%	62.5%	12.5%	16.7%	100%
S3	1	11	9	3	24
%	4.2%	45.8%	37.5%	12.5%	100%
S4	6	13	5	0	24
%	25.0%	54.2%	20.8%	0.0%	100%
Total	9	52	23	12	96
%	9.4%	54.2%	24.0%	12.5%	100%

Multinomial logistic regression shows that a significant effect on the remission of candidiasis was observed with S3 and S1. The order and sequence of treatments had no influence in the results (Table 3).

**Table 3:** Effect of source factors on remission of candidiasis

	Num DF	Den DF	F Value	Pr > F
Baseline and washout	3	61	4.51	0.0064
Treatment	3	61	4.44	0.0069
Order	3	61	0.52	0.6691
Sequence	3	20	0.74	0.5412

Num DF and Den DF: Degrees of freedom used in determining the F values.

Pr > F: p-value associated with the F value of the statistical test. The null hypothesis the specified canonical correlations are equal to zero is evaluated with regard to this p-value. The null hypothesis is rejected if the p-value is less than the specified alpha level (0.05).

F Value - F Value - Test the hypothesis that both canonical correlations are equal to zero in the sample.

Patients’ satisfaction results are show in Table 4. In question 1, the effects of different solutions (p=0.20) and inflammation level (p=0.57) did not influence patient’s responses, and the inflammation × solution interaction could not be assessed due to lack of variability of the responses. Regarding questions 2 (solution: p=0.9; inflammation: p=0.8; interaction: p=0.9), 3 (solutions: p=0.2; inflammation: p=0.2; interaction: p=0.5), 5 (solutions: p=0.7; inflammation: p=0.8; interaction: p=0.08), and 6 (solutions: p=0.6; inflammation: p=0.5; interaction: p=0.2), results were also non-significant. Regarding question 4, only the effect of inflammation could be assessed, which was also non-significant (p=0.6).

**Table 4:** Percentage of patients for score 0 or 10 for each question and treatment

	Score	S1	S2	S3	S4
Q1	0	1.6%	1.6%	6.2%	6.2%
	10	98.4%	98.4%	93.8%	93.8%
Q2	0	15.6%	20.3%	10.9%	7.8%
	10	84.3%	79.7%	89.1%	92.2%
Q3	0	31.3%	20.3%	17.2%	18.8%
	10	68.8%	79.7%	82.8%	81.3%
Q4	0	4.7%	0%	1.6%	0%
	10	95.3%	100%	98.4%	100%
Q5	0	9.4%	6.3%	9.4%	6.3%
	10	90.6%	93.8%	90.6%	93.8%
Q6	0	6.3%	6.3%	9.4%	6.3%
	10	93.8%	93.8%	90.6%	93.8%

**Figure 4:**
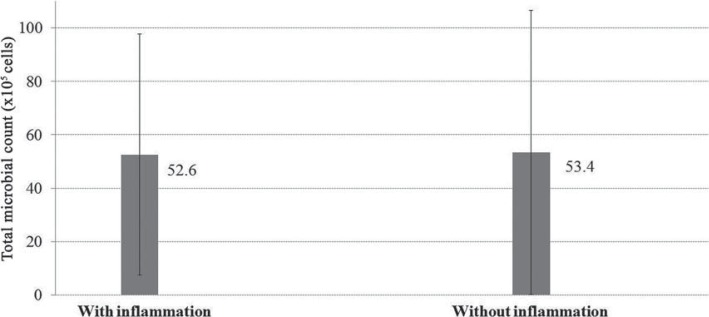
Mean total microbial count (×105 cells, ±SD) of the groups with and without inflammation (p=0.075)

**Figure 5:**
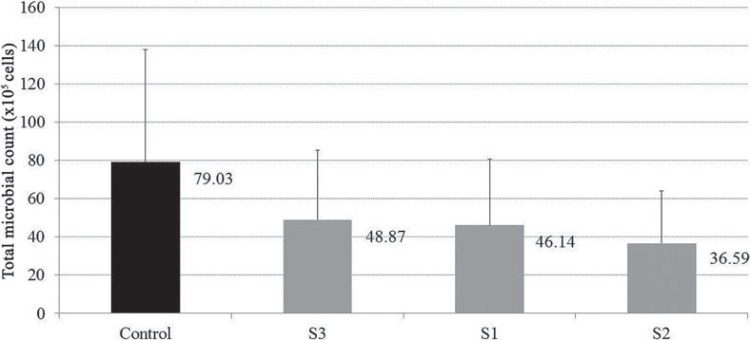
Mean total microbial count (×105 cells, ±SD) of the 43 evaluated species dentures after treatment (Different colors indicate significant differences; P=0.041)

For DNA-Checkerboard hybridization results, no differences were found in the amount of total microorganism count between groups with and without candidiasis (p=0.75; Figure 4) or in the interaction between inflammation and solution (p=0.98). Total microorganisms counts were similar after use of S1, S2, and S3 solutions and lower than S4 (Figure 5).

The effects of solutions on individual microorganism count are shown in Table 5. S1 and S3 showed intermediate efficacy between S2 and S4 against fifteen microorganisms (*C. tropicalis; C. krusei; S. sanguinis; S. oralis; S. mutans; P. intermedia; L. casei; C. rectus; A. actinomycetemcomitans serotype b; S. moorei; S. constellatus; P. putida; P. micra; P. anaerobios; K. penumoniae*). S2 and S3 were efficient against *C. dubliniensis* and *P. melaninogenica*. S1 and S2 caused significant reductions for *F. nucleatum, S. pasteuri, P. endodontalis, N. mucosa*, and *F. periodonticum*. S3 was effective against *P. aeruginosa.* S1 and S3 caused a mild reduction in the count of *E. coli* and *A. actinomycetemcomitans* serotype a, against which S1 was more efficient.

**Table 5:** Mean, median, lower, and upper quartiles of microbial counts (×105 ±SD) and respective p-values after treatments

	S1					S2					S3					Control					
	Mean	±SD	Lower	Median	Upper	Mean	±SD	Lower	Median	Upper	Mean	±SD	Lower	Median	Upper	Mean	±SD	Lower	Median	Upper	p-value
			quartile		quartile			quartile		quartile			quartile		quartile			quartile		quartile	
*C. tropicalis*	0.61	1.34	0	0ab	0	0.33	1.05	0	0b	0	0.68	1.44	0	0ab	0	1.37	1.77	0	0a	3.51	0.00
*C. krusei*	0.45	1.20	0	0ab	0	0.27	0.91	0	0b	0	0.36	1.03	0	0ab	0	1.03	1.66	0	0a	3.45	0.00
*C. glabrata*	0.42	1.20	0	0b	0	0.27	0.93	0	0b	0	0.44	1.19	0	0b	0	1.06	1.71	0	0a	3.26	0.02
*C. dubliniensis*	0.50	1.32	0	0ab	0	0.26	1.03	0	0b	0	0.42	1.21	0	0b	0	0.89	1.70	0	0a	0	0.02
*C. albicans*	0.36	1.13	0	0a	0	0.26	1.02	0	0a	0	0.21	0.83	0	0a	0	0.64	1.40	0	0a	0	0.12
*V. parvula*	0.27	1.07	0	0b	0	0.40	1.27	0	0b	0	0.65	1.63	0	0b	0	0.93	1.81	0	0a	0	0.02
*T. denticola*	1.36	2.11	0	0a	3.96	1.24	2.10	0	0a	3.36	1.59	2.21	0	0a	4.1	1.77	2.16	0	0a	39.3	0.43
*T. forsythia*	0.96	1.75	0	0a	2.54	0.45	1.29	0	0a	0	0.63	1.39	0	0a	0	1.13	1.89	0	0a	3.53	0.07
*S. sobrinus*	1.38	2.02	0	0a	3.98	1.04	1.92	0	0a	3.75	1.40	2.05	0	0a	4.1	2.32	2.18	0	0a	4.11	0.43
*S. sanguinis*	1.01	1.87	0	0ab	0	0.49	1.42	0	0b	0	1.23	2.19	0	0ab	4.1	1.45	2.18	0	0a	4.11	0.01
*S. salivarius*	1.69	2.25	0	0b	4.19	1.52	2.68	0	0b	3.63	2.51	2.24	0	3.52b	4.37	3.53	2.40	0	3.86a	5.26	0.00
*S. pasteuri*	0.87	1.75	0	0b	0	0.57	1.55	0	0b	0	1.15	1.86	0	0ab	3.38	2.03	2.42	0	0a	3.93	0.00
*S. parasanguinis*	1.82	2.51	0	0b	4.07	1.50	2.52	0	0b	3.58	2.02	2.39	0	0ab	4.16	3.32	2.01	3.13	3.84a	4.81	0.00
*S. oralis*	1.11	1.87	0	0ab	3.19	0.68	1.52	0	0b	0.00	1.18	1.90	0	0ab	3.31	1.89	2.14	0	0a	4.13	0.01
*S. mutans*	0.57	1.52	0	0b	0	0.35	1.10	0	0b	0	0.71	1.65	0	0ab	0	1.04	1.84	0	0a	2.61	0.04
*S. moorei*	1.82	1.71	0	0b	0	1.50	1.50	0	0b	0	2.02	1.50	0	0b	0	3.32	2.43	0	0a	2.82	0.00
*S. mitis*	2.02	2.62	0	0b	4.27	1.69	2.92	0	0b	3.47	2.39	2.24	0	3.46b	4.31	3.95	2.41	3.28	4.37a	5.47	0.00
*S. gordonii*	1.72	2.58	0	0b	3.89	1.29	2.71	0	0b	0	1.40	2.02	0	0b	3.42	2.89	2.51	0	3.56a	5.09	0.00
*S. constellatus*	0.72	1.70	0	0ab	0	0.50	1.36	0	0b	0	0.85	1.72	0	0ab	0	1.44	2.08	0	0a	3.88	0.00
*S. aureus*	2.11	2.77	0	0a	4.56	1.72	2.37	0	0a	4.28	1.72	2.27	0	0a	4.09	1.78	2.36	0	0a	4.29	0.35
*P. putida*	1.08	1.97	0	0ab	0	0.63	1.56	0	0b	0	1.06	1.79	0	0ab	3.17	1.62	2.26	0	0a	3.92	0.02
*P. nigrescens*	0.96	1.85	0	0b	0	0.82	1.73	0	0b	0	0.97	1.78	0	0b	0	2.00	2.09	0	0a	4.02	0.00
*P. micra*	1.19	2.08	0	0ab	3.6	0.86	1.82	0	0b	0	1.10	2.05	0	0ab	0	1.67	2.22	0	0a	3.67	0.01
*P. melaninogenica*	1.93	2.38	0	0ab	4.43	1.57	2.36	0	0b	4.89	1.59	2.15	0	0b	3.93	2.78	2.49	0	4.23a	4.74	0.02
*P. intermedia*	0.90	1.82	0	0ab	0	0.57	1.53	0	0b	0	0.85	1.63	0	0ab	0	1.66	2.03	0	0a	3.81	0.01
*P. gingivalis*	1.07	2.02	0	0a	0	0.54	1.43	0	0b	0	0.93	1.81	0	0ab	0	1.38	2.10	0	0a	3.6	0.01
*P. endodontalis*	0.98	2.10	0	0b	0	0.82	1.74	0	0b	0	1.29	2.01	0	0ab	3.05	2.08	2.16	0	2.82a	3.92	0.00
*P. anaerobios*	0.72	1.61	0	0ab	0	0.26	1.02	0	0b	0	0.52	1.40	0	0b	0	0.96	1.88	0	0a	0	0.02
*P. aeruginosa*	1.14	1.84	0	0b	3.39	1.36	3.81	0	0b	0	0.52	1.39	0	0c	0	1.91	2.14	0	0a	4.08	0.00
*N. mucosa*	1.11	1.97	0	0b	3.24	0.97	1.88	0	0b	0	1.47	2.09	0	0ab	3.75	2.30	2.20	0	3.41a	4.01	0.00
*M. salivarium*	2.66	2.46	0	4.01a	4.67	2.28	2.45	0	0a	4.81	2.54	2.44	0	3.8a	4.7	3.10	2.28	0	4.23a	4.83	0.17
*L. casei*	0.84	1.91	0	0ab	0	0.57	1.41	0	0b	0	1.30	1.99	0	0ab	3.18	1.58	2.15	0	0a	3.91	0.00
*K. pneumoniae*	1.37	2.20	0	0ab	3.78	0.86	1.81	0	0b	0	1.49	2.10	0	0ab	3.71	2.10	2.35	0	0a	4.27	0.00
*F. periodonticum*	0.87	1.66	0	0b	0.00	0.70	1.67	0	0b	0	1.25	1.90	0	0ab	3.27	1.79	2.13	0	0a	3.92	0.00
*F. nucleatum*	1.82	2.23	0	0b	4.12	1.97	2.54	0	0b	4.5	2.30	2.47	0	0ab	4.65	2.96	2.34	0	4.12a	4.75	0.00
*E. faecalis*	0.80	1.65	0	0b	0	0.77	1.71	0	0b	0	0.81	1.71	0	0b	0	1.79	2.10	0	0a	3.89	0.01
*E. corrodens*	1.34	2.01	0	0b	3.81	1.42	2.17	0	0b	3.6	1.20	1.96	0	0b	3.41	2.27	2.25	0	3.25a	4.25	0.03
*E. coli*	0.62	1.45	0	0b	0	0.82	1.75	0	0b	0	1.02	1.79	0	0ab	2.82	1.56	1.98	0	0a	3.83	0.03
*C. rectus*	0.19	0.87	0	0bc	0	0.14	0.78	0	0c	0	0.49	1.43	0	0ab	0	0.78	1.66	0	0a	0	0.00
*C. gingivalis*	1.19	1.94	0	0b	3.66	1.29	2.19	0	0ab	3.78	1.37	2.08	0	0ab	3.69	2.21	2.12	0	3.24a	4.04	0.04
*B. fragilis*	0.45	1.20	0	0a	0	0.54	1.34	0	0a	0	0.73	1.46	0	0a	0	1.05	1.70	0	0a	3.24	0.05
*Aa serotype a*	0.91	1.59	0	0b	2.92	1.14	1.88	0	0ab	3.34	1.36	1.83	0	0ab	3.38	1.82	1.96	0	0a	3.63	0.02
*Aa serotype b*	0.57	1.32	0	0ab	0	0.27	0.95	0	0b	0	0.61	1.36	0	0a	0	0.83	1.57	0	0a	0	0.04

Aa:*Aggregatibacter actinomycetemcomitans*

## Discussion

The association of mechanical and chemical methods have been recommended for the control of denture biofilm formation, thus avoiding the development of inflammatory processes^7^ ^,^ ^14^ ^,^ ^16^ ^,^ ^22^. The most commonly used chemical solution is NaClO, however it can cause deleterious effects to the denture when used at 1% or 0.5% concentrations^4^ ^,^ ^17^ ^,^ ^19^ ^,^ ^20^. Therefore, the assessment of NaClO at lower concentrations, as well as of new chemicals, is needed to help clinicians and patients find more suitable solutions.

The first null hypothesis was partially accepted. S1 was similar to S2 in the ability to remove biofilm, followed by S3. Results showed that S1, S2, and S3 solutions significantly reduced total (pool) and individual microbial counts of target species. All treatments were better than control (S4). Previous studies have shown that the immersion of dentures in 0.5% sodium hypochlorite is effective in biofilm removal^2^ and in the reduction of microorganism count^22^ ^,^ ^23^. These results demonstrate that lower concentrations of sodium hypochlorite or the use of *R. communis* are an effective solution against biofilm formation and for microorganism reduction and an alternative for hypochlorite at 0.5%, which have been recommended from other studies^8^ ^,^ ^22^ ^,^ ^23^. Percentages of biofilm and the microorganism count were not influenced by the presence or absence of inflammation. However, it is still necessary to evaluate the adverse effects of 0.25% NaClO and 10% *R. communis* (S3) on the acrylic resin of the denture. In the literature, only one study evaluated the surface roughness with the same solutions, which demonstrated clinically acceptable values, once they were below of 0.2 μm^4^.

*R. communis* was used in this investigation once it shows antimicrobial properties similar to NaClO when used in root canals with necrotic lesions^11^. In addition, it is also biocompatible^9^ and has detergent properties. There are studies evaluating the efficiency of *R. communis* solution in achieving complete denture hygiene, although experimental designs are diverse and results are inconclusive^2^ ^,^ ^10^ ^,^ ^19^ ^,^ ^20^ ^,^ ^22^ ^,^ ^23^.

S3 solution showed mild results in biofilm removal. Andrade, et al.^2^ (2014) reported similar ratios between 2% *R. communis* and alkaline peroxide, but different ratios from 1% NaClO. Based on those previous findings, this study evaluated a higher *R. communis* concentration (10%), as an attempt to reach similarity with NaClO. Although biofilm reduction with S3 was lower than with hypochlorite, S3 presented better results than the control. Thus, *R. communis* can be considered an alternative to hypochlorite for allergic patients, once it also presents biocompatibility with living tissues^6^ ^,^ ^9^.

When the effects of the solutions on individual microorganisms were evaluated, S3 showed similar results to hypochlorite (S1 and S2) against *C. glabrata, V. parvula, S. salivarius, S. mitis, S. gordonii, S. moorei, P. nigrescens, E. faecalis*, and *E. corrodens*. S3 had also the same effect as S2 against *P. anaerobius* and *C. dublinienses;* S3 was more effective than S1 and S2 against *P. aeruginosa*. Against other microorganisms such as *C. tropicalis, C. krusei, E. coli*, and *S. mutans*, S3 showed results that were mild, less effective than both concentrations of hypochlorite but more effective than saline. It is noteworthy that no difference between treatments was found in the count of *C. albicans* and *S. aureus*, two important species found in the denture biofilm. Studies have reported that the detergent properties of *R. communis* cause damage to the cell wall, resulting in loss of the constituents of cytoplasm and subsequent cell death^12^ ^,^ ^30^. These action mechanisms however need to be further investigated.

The use of saline as a control substance resulted in the highest percentage of biofilm among the evaluated solutions. This result was expected and confirmed the findings of Andrade, et al.^2^ (2014). However, the act of brushing followed by immersion in saline reduced the amount of biofilm when compared to baseline, confirming the efficiency of mechanical brushing found in previous studies^16^. However, while brushing efficiently cleans the denture surface from solid particles, it is not enough for eliminating microorganism from micro-irregularities of denture surfaces. Thus, the association of mechanical and chemical methods is recommended for proper denture hygiene^7^ ^,^ ^22^. This effective association explains the significantly reduced counts of microorganisms in dentures treated with antimicrobial solutions which, in turn, cause the dissolution of the biofilm organic matrix.

Regarding the remission of candidiasis, the immersion in 10% *R. communis* and 0.25% sodium hypochlorite results in lower scores of inflammation than 0.5% sodium hypochlorite. S3 solution had the best results for remission of candidiasis in 50% of participants. This finding corroborates Pinelli, et al.^18^ (2013), in whose study a castor oil based solution improved clinical symptoms of candidiasis in older adult patients, similarly to the effect of Miconazole. In this study, NaClO solution was more efficient at 0.25% than at 0.5% concentration. This result is contrary to most studies on the NaClO ability of biofilm removal and antimicrobial action. Perhaps an allergic and/or irritant action caused by residual waste solutions employed in acrylic resin, could influence the degree of inflammation seen on the oral mucosa of the palate and/or alveolar ridge^3^. A limitation of this study was that residual effect of NaClO on the acrylic resin was not evaluated. Moreover, clinical trials evaluating the irritating action of hypochlorite on the oral mucosa and long-term evaluation are necessary.

Patients with and without denture stomatitis participated in this study in order to determine whether the analyzed solutions can be used for cleaning of dentures giving preventive and curative actions against candidiasis.

Results of the questionnaire showed that S1, S2, and S3 had similar patient approval than saline, rejecting the second null hypothesis. This demonstrates that the use of these solutions did not cause any inconvenience to participants, which would have a positive influence in patient compliance with prostheses home care. However, this is in contrast with some studies that emphasize malodor and unpleasant taste of NaClO as one of its disadvantages.

Finally, this study reinforces that 10% *R. communis* and 0.25% NaClO solutions can be used as denture cleanser replacing the 0.5% NaClO as auxiliary agent for the mechanical method of brushing. Other studies should be used in addition, evaluating these solutions to reinforce their viability of use such as research on biomechanical analysis.

## Conclusion

Tested solutions caused significant reduction in biofilm percentage, in total microorganisms count, and were approved by the participants. *R. communis* solution and 0.25% NaClO were effective in the remission of candidiasis. 0.25% sodium hypochlorite and *R. communis* can be indicated as a denture cleanser.
